# The Mechanism by which 146-N-Glycan Affects the Active Site of Neuraminidase

**DOI:** 10.1371/journal.pone.0135487

**Published:** 2015-08-12

**Authors:** Pi Liu, Zhonghua Wang, Lijie Zhang, Dongmei Li, Jianping Lin

**Affiliations:** State Key Laboratory of Medicinal Chemical Biology and College of Pharmacy, Nankai University, Tianjin, 300071, China; Wake Forest University, UNITED STATES

## Abstract

One of the most conserved glycosylation sites of neuraminidase (NA) is 146-N-glycan. This site is adjacent to the 150-cavity of NA, which is found within the active site and thought to be a target for rational drug development against the antiviral resistance of influenza. Here, through a total of 2.4 μs molecular dynamics (MD) simulations, we demonstrated that 146-N-glycan can stabilize the conformation of the 150-loop that controls the volume of the 150-cavity. Moreover, with 146-N-glycan, our simulation result was more consistent with crystal structures of NAs than simulations conducted without glycans. Cluster analysis of the MD trajectories showed that 146-N-glycan adopted three distinct conformations: monomer-bridged, dimer-bridged and standing. Of these conformations, the dimer-bridged 146-N-glycan was the most stable one and contributed to stabilization of the 150-loop conformation. Furthermore, our simulation revealed that various standing conformations of 146-N-glycan could block the entrance of the binding pocket. This result was consistent with experimental data and explained the relatively low activity of inhibitors with flexible substituents toward the 150-cavity. Together, our results lead us to hypothesize that rigid and hydrophobic substituents could serve as better inhibitors targeting the 150-cavity.

## Introduction

Influenza viruses have extraordinary virulence, causing severe respiratory infection and even death. Every decade or so, a dangerous new strain appears and poses a threat to the public health [1]. Of the three types of influenza virus, type A infects a wide range of avian and mammalian species. Additionally, type A can be further classified into subtypes according to the serological reactivity of its surface glycoprotein antigens, hemagglutinin (HA) and neuraminidase (NA) [2]. Of all the serotypes, there are nine for NA (N1 to N9) and sixteen for HA (H1 to H16) found in avian and mammalian hosts [3]. The nine NA alleles have been divided into two groups based on phylogenetic analyses of the species (group-1: N1, N4, N5, N8; group-2: N2, N3, N6, N7, N9) [4]. Of these alleles, N1 and N2 are responsible for viral pandemics and recurrent annual epidemics in humans [5]. The influenza virus NA is a tetramer protein that catalyzes the cleavage of terminal α-ketosidically linked sialic acids from a large variety of glycoproteins, glycolipids, and oligosaccharides [6, 7]. NA plays an important role in the final stages of influenza virus infection, removing sialic acid from both the infected cell surfaces and the newly formed virus. Simultaneously, NA facilitates the release of progeny viruses and thus furthers the spread of infection [8]. Given its importance in altering the progression of influenza infection, NA is an attractive target for structure-based antiviral drug design.

To date, only four anti-flu drugs have been approved by the Food and Drug Administration (FDA). Two (amantadine and rimantadine) target the M2 ion channel of the influenza virus [9]. The remaining two, oseltamivir (Tamiflu) and zanamivir (Relenza), both inhibit NA [10, 11]. However, because these drugs are widely used, various NA subtypes have demonstrated resistance to the M2 channel inhibitors amantadine and rimantadine [12]. Fortunately, oseltamivir and zanamivir are still effective against new viruses. However, NA also faces selection pressure as mutations occur. Once drug-resistant strains arise, they will likely lead to the breakout of a novel flu, causing greater global public health concerns. Thus, to develop new drugs for NA, it is vital to proactively identify potential drug-resistance sites and identify the optimal binding modes of current drugs with NA.

Adjacent to the sialic acid binding site, a difference occurs between group-1 and group-2 NAs. In the crystal structure of NAs, the 150-loop has two distinct conformations, one open and one closed [13]. The majority of the crystal structures of group-1 NAs contain a 150-cavity that is rarely found in the crystal structures of group-2 NAs [13]. It has been hypothesized that targeting the 150-cavity may allow for the development of new antiviral agents with increased specificity and potency against group-1 enzymes [14]. Earlier computational studies revealed that a key salt bridge (D147-H150) predominantly controlled the closed conformation of the 150-cavity in group-2. The lack of this salt bridge provides flexibility to the 150-loop, which could be responsible for the open conformation in group-1 [13]. In these simulations, the open form of the 150-loop was noted as the main conformation in group-1 and was proposed as a new potential target for drug design [14]. The closed conformation previously observed in group-2 structures suggested that a slow conformational change may occur upon inhibitor binding to the NA of group-1 [15, 16]. However, in the crystal structure 3NSS (group-1 NA), the closed conformation can also be observed without an inhibitor binding in the pocket [17]. Therefore, we hypothesize that there are additional factors that contribute to stabilizing the 150-loop of the NA in group-1. After investigating the 150-loop of NA, we found that none of these simulations considered the possible effects of N-glycan, which is located on residue N146 adjacent to the 150-loop.

In the past decades, numerous studies have demonstrated that glycosylation on HA and NA of influenza strains can affect host specificity, infectivity and virulence by altering the biological properties of HA and NA [18]. During the 1918 pandemic influenza virus, different N-glycan profiles may have caused viral resistance to proteinase digestion and lead to increased infectivity [18]. The glycosite located near the enzymatic active site on ASN146 of the NA N-terminus close to the 150-loop (residues 147–152) is highly conserved across approximately all strains [19]. To reveal the effects of the N-glycan on the conformational stability of the 150-loop and volume of the 150-cavity, we examined the flexibility of the 150-loop with and without 146-N-glycan using molecular dynamics (MD) simulations of the 09N1 crystal structure as well as other available structures of N1 and N2 alleles derived from human clinical samples. Additionally, we studied how 146-N-glycan affected the 150-cavity when the 150-loop was in an open conformation. Our results indicate that the 146-N-glycan could stabilize the 150-loop conformations and preferentially prevent transformation of the 150-loop from closed to open in both N1 and N2 enzymes. Conversely, the volume of the 150-cavity and the entrance to the 150-cavity could also be controlled and affected by the 146-N-glycan, which helps to explain the low activity of inhibitors targeting the 150-cavity of NA enzymes.

## Results and Discussion

### Molecular Dynamics Simulations

To probe the effect of the N-glycan on the conformation of the 150-loop, we performed six separate 100 ns MD simulations with AMBER 12 [20] for three tetrameric NA enzymes with and without a 146-N-glycan: (1) A/Tokyo/3/67, a seasonal human H2N2 virus (N2, PDB ID: 1NN2) [21]; (2) A/Vietnam/1203/04, an avian-derived H5N1 virus isolated from a human (VN04N1, PDB ID: 2HTY) [14]; (3) A/California/04/2009, an H1N1 virus isolated early during the 2009 pandemic (09N1, PDB ID: 3NSS) [17]. The 1NN2 structure exclusively contained a salt bridge stabilizing the closed conformation of the N2 sample. The 2HTY structure was selected to represent an open conformation of N1 samples. The 3NSS structure was selected to represent a closed conformation of N1 samples.

Simulating the homo-tetramer configuration of NA led to the equivalent of approximately 0.5 μs (400 ns) of sampling for each NA monomer while simultaneously incorporating neighboring subunit (monomer) structural effects. The tetramer systems and individual monomer chains exhibited stability over 100 ns in backbone root mean square deviation (RMSD) plots (S1 Fig). Additionally, the experimental and simulation-derived B-factors were consistent (S2–S4 Figs).

### 146-N-glycan stabilizes predominant l50-loop conformations and prevents conformational transformation in both N1 and N2

**Fig 1:**
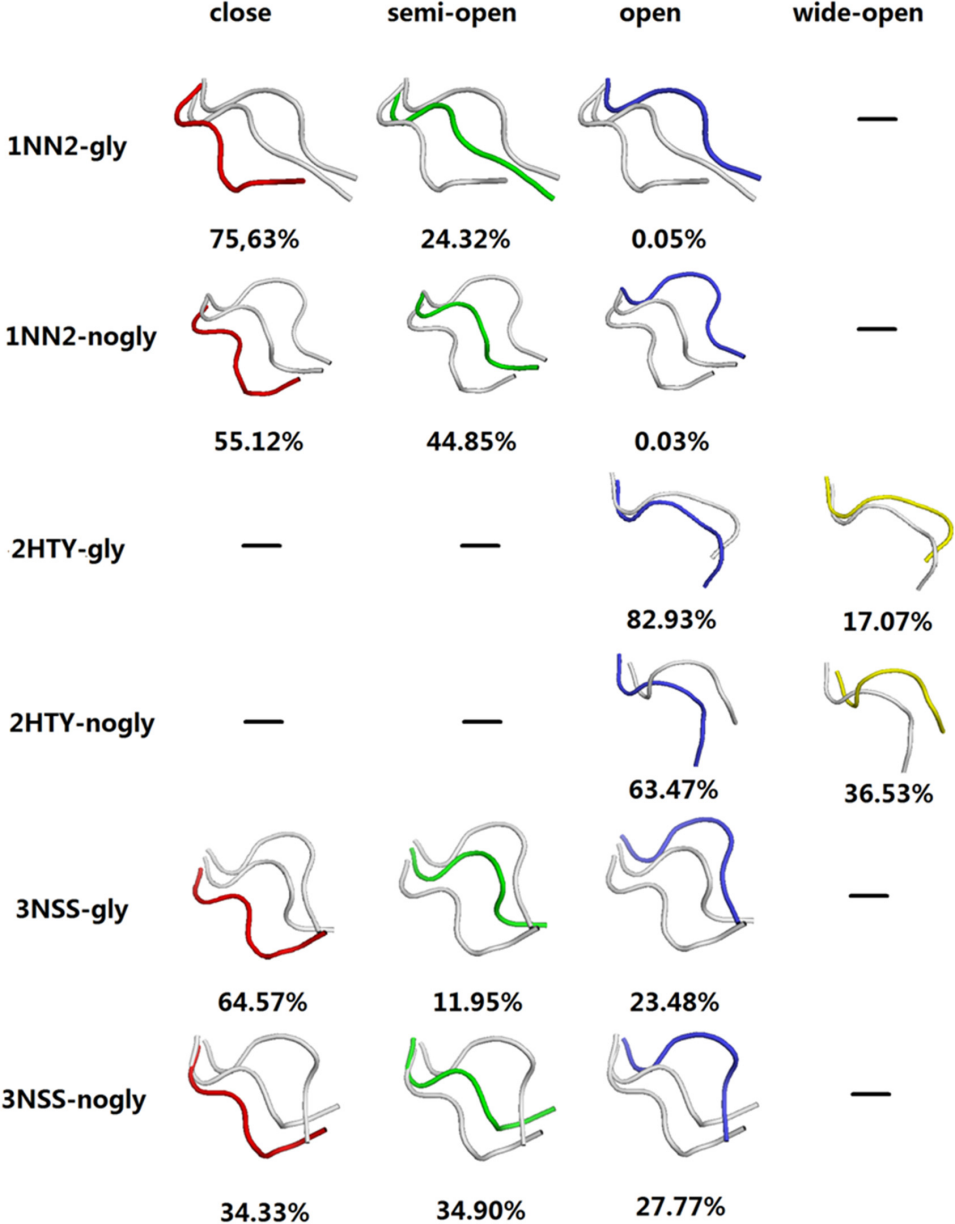
Conformation clusters of 150-loop from the six MD trajectories. Loop clusters colored red, green, blue and yellow represent close, semi-open, open and wide-open conformations, respectively. “-” represent no such conformation. “-gly” means with glycan, “-nogly” means without glycan.

Our simulations revealed that 146-N-glycan stabilizes predominant 150-loop conformations and prevents the conformational transformation from closed to open in both N1 and N2 (Fig 1). Surprisingly, the pandemic 09N1 with 146-N-glycan maintained its preferential conformation with a closed 150-cavity in normal solution dynamics. This finding is in contrast with both 09N1 without 146-N-glycan simulation (Fig 1) and simulations of other groups [13, 14]. All six systems (three with the 146-N-glycan and three without) were simulated using 100 ns MD simulations. The snapshots of the four chains obtained every 40 ps were clustered with cpptraj analysis tool of AMBER 12 and then used to count the proportion of open versus closed conformations. The epsilon value was set to 2.0 Å. Crystal structures of 3NSS and 2HTY were selected as references for the closed and open 150-loop N1 conformations, respectively. The 1NN2 crystal structure was used as a reference for the closed 150-loop conformation of N2, whereas the semi-open, open and wide-open conformations of NA were defined with a cluster conformation whose loop RMSD (residues 148–151) was approximately 2.0 Å, 4.0 Å and 6.0 Å different from that of 1NN2, respectively. These conformational clusters do not correlate with the ability of ligand binding to the 150-cavity. However, the conformational distribution of the 150-loop might affect the selectivity of the NA inhibitors (see S5 Fig and S1 Table for a detailed discussion).

Cluster analysis of the 150-loop in the six MD trajectories is presented in Fig 1. For the 09N1 protein 3NSS with 146-N-glycan, 64.6% of the conformations of the 150-loop in trajectories were closed, whereas 35.4% were open (semi-open and open). This observation could explain why the crystal structure of 3NSS was in the closed 150-cavity conformation, as the preferential conformation of 3NSS in solution is closed. When simulating 3NSS without 146-N-glycan, approximately 62.7% of the conformations of 150-loop were in the open form (semi-open and open), which was consistent with simulation from other groups [13, 15]. This result indicates that in simulating influenza A NA proteins, glycan exerts an important and non-negligible effect. For the H5N1 protein 2HTY, all 150-loop conformations both with and without 146-N-glycan were in the open (open and wide open) form. The main difference in these molecules between those with glycan and those without is that simulating with glycan led to approximately 20% more regular open 150-loop conformations. Approximately 1/3 of 09N1 and 100% of H5N1 150-loop conformations were open, which provides a structural understanding for the fact that compounds designed to target the 150-cavity can inactivate both 09 H1N1 and H5N1 viruses. In the simulation of the N2 protein 1NN2 with 146-N-glycan, 75.6% of the 150-loop conformations remained closed. For 1NN2 without 146-N-glycan, however, the percentage of closed conformations was 55.1%. Based on the six trajectories, for both N1 and N2 with 146-N-glycan, greater than 20% more of the initial conformational form (whether open or closed) was noted for both N1 and N2 with 146-N-glycan compared with N1 and N2 without 146-N-glycan. This result indicates that glycan can stabilize the predominant 150-loop conformations and prevent the conformational transformation from closed to open in both N1 and N2. As previous simulations have revealed, the 150-loop may not exhibit high levels of flexibility in the presence of glycan [13–15].

### Monomer-bridged, dimer-bridged and standing conformations of the 146-N-glycan

**Fig 2:**
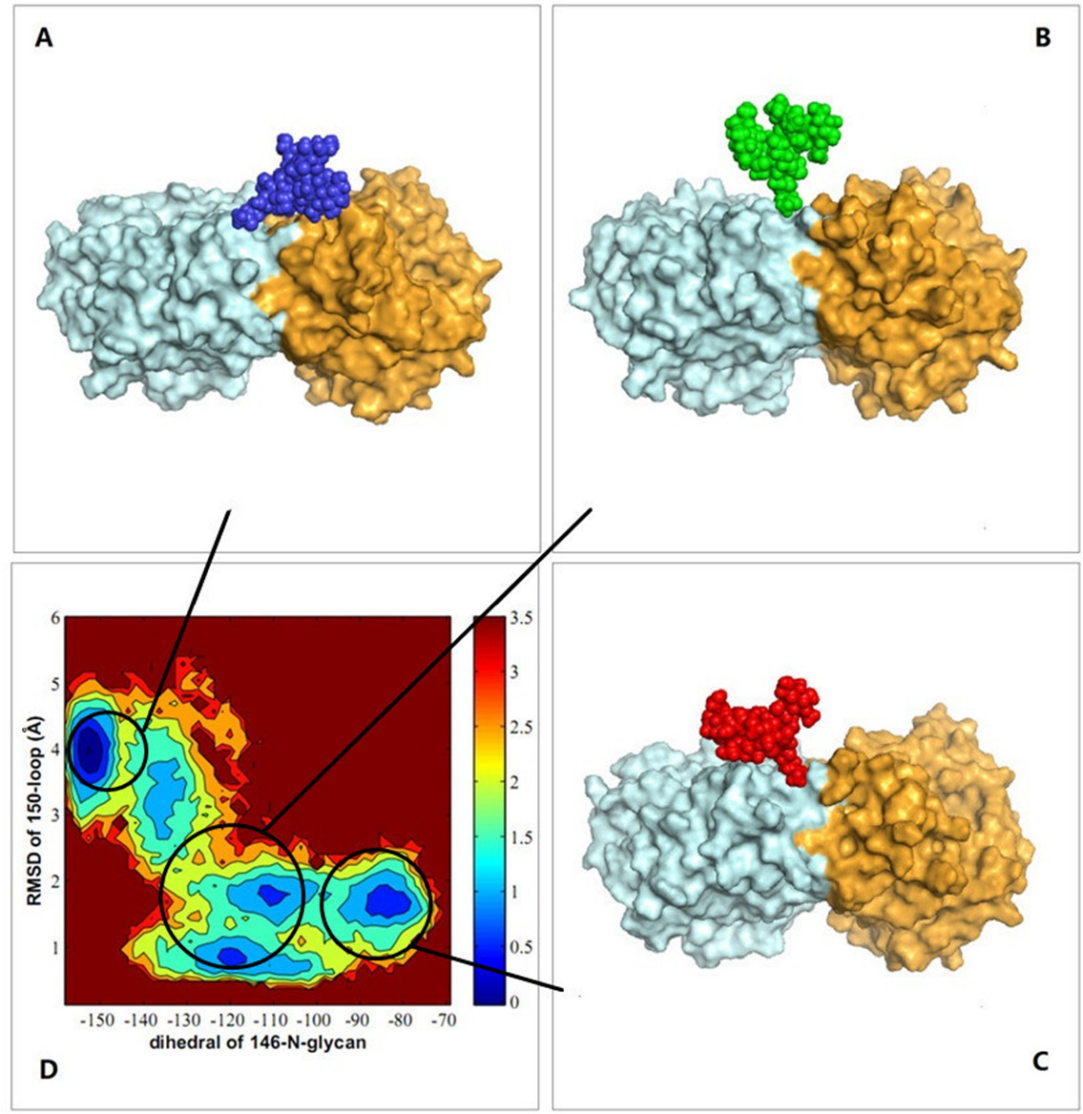
Representative conformations of 146-N-glycan. Glycan is in CPK representation, protein chain is in surface representation. (A) Dimer-bridged conformation. (B) Standing conformation. (C) Monomer-bridged conformation. (D) A contour plot of conformational density distribution, ρ, represented by the molecular potential,-*RTln(ρ)* in units of kcal/mol.

**Fig 3:**
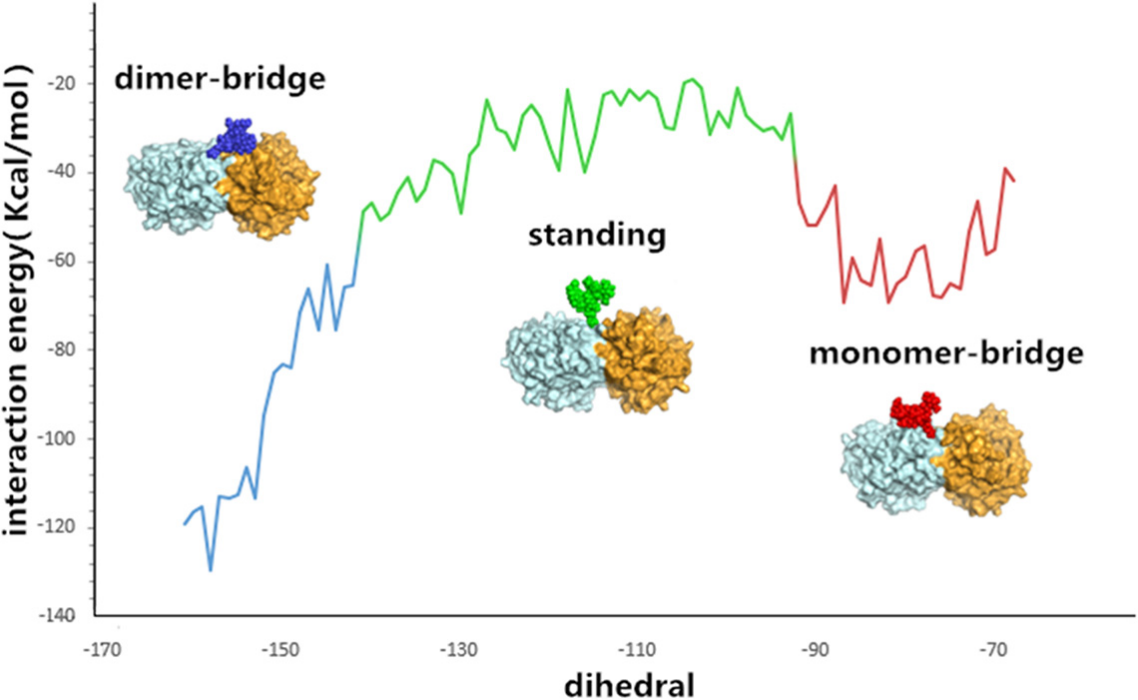
The relationship of glycan interaction energies and the dihedral angle of 146-glycan. Interaction energy was defined between each glycan and the other parts of tetramer of 3NSS. Dihedral angle of 146-N-glycan was plane angle between plane defined by mass center of glycan, S145: CA, L223: CA and plane defined by S145: CA, L223: CA, P301: CA. Glycan is in CPK representation, protein chain is in surface representation. The blue, green, red curves represent dimer-bridge, standing and monomer-bridge conformations.

To study the mechanism by which the 146-N-glycan stabilizes the conformations of the 150-loop, conformations of the 146-N-glycan of three systems with glycan were clustered. A relative, stable reference plane (defined by CA atom of S145 and two β-sheet end CA atoms which have the following B-factors: S145:CA = 11.77, L223:CA = 8.07 and P301:CA = 8.10) was chosen to define the dihedral angle of the 146-glycan (S6 Fig). The glycan conformations on the protein were divided into three clusters: monomer-bridged, standing and dimer-bridged (Fig 2). Monomer-bridged is the conformation in which the glycan attaches to the same chain to which 146-N-glycan is bound. Dimer-bridged is the conformation in which the glycan bridges to the adjacent chain. The standing conformation is that in which the glycan stands perpendicular to the dimer interface, as presented in Fig 2. Both monomer-bridged and dimer-bridged conformations form structures that are more stable than those formed by the standing conformation by interacting with a monomer or adjacent chain or glycans. In three MD systems, the percentages of conformations among the clusters were different, as presented in Table 1. In 1NN2, 94.30% of the glycan conformations were standing or monomer-bridged. In 2HTY, 94.25% of the glycan conformations were dimer-bridged or standing. In 3NSS, 73.78% of the glycan conformations were monomer-bridged or standing. These results indicate that the closed 150-loop conformation correlates with the monomer-bridged glycan conformation, whereas the dimmer-bridged glycan conformation is related to the open 150-loop conformation. However, when glycan is in the standing conformation, the 150-loop conformation can be either open or closed. This connection can be clearly demonstrated with a correlation map of the 150-loop conformations and glycan conformations of 3NSS, which contained all three 150-loop conformations (closed, semi-open and open; (Fig 2)). In Fig 2D, the conformational density distribution of each snapshot extracted from the trajectory files obtained every 10 ps is presented. The 150-loop RMSD (y-axis) and dihedral of 146-N-glycan (x-axis, dihedral angle between plane defined by mass center of glycan, S145:CA, L223:CA and plane defined by S145:CA, L223:CA, P301:CA) present the conformational relationship of the 150-loop and 146-N-glycan. The reference conformation of the 150-loop is closed. For the 150-loop RMSD, 0~2 Å represents the closed conformation, 2~4 Å represents the semi-open conformation, and greater than 4 Å represents the open conformation. For the dihedral of 146-N-glycan, -160 ~ -140° represents the dimer-bridged conformation, -140 ~ -90° represents the standing conformation, and -90~ -70° represents the monomer-bridged conformation. Three distinct clusters are presented in Fig 2D that correlate with the three distinct glycan conformations shown in Fig 2A–2C. Through comparisons with the 150-loop RMSD, we found that the monomer-bridged conformation correlated with the closed 150-loop, and the dimer-bridged conformation correlated with the open 150-loop. The standing conformation correlated with both closed and open 150-loop forms. To further study the stability of the system, interaction energies between 146-N-glycan and the other parts of 3NSS NA were also calculated using AMBER 12 software (Fig 3; calculation details were provided in S7 Fig and S1 Text). As shown in Fig 3, the binding energies of monomer-bridged conformations were approximately 20 to 40 kcal/mol reduced compared with standing conformations. Additionally, the dimer-bridged conformation energies were approximately 40 to 80 kcal/mol lower than those of the standing conformations, which indicates that monomer-bridged and dimer-bridged conformations are more stable. Specifically, the dimer-bridged conformation, which displayed the lowest binding energy and correlated with 150-loop open form, was noted as the most stable conformation. These results can be explained by a model in which no transformation from the open to the closed 150-loopis noted when simulating the H5N1 protein (2HTY), whereas a small number of closed to open form transformations occur when simulating using the closed conformation (3NSS, 1NN2). Our findings indicate that 146-N-glycan can stabilize predominant l50-loop conformations and prevent conformational transformation in both N1 and N2.

**Table 1:** Percentages of three types of 146-N-glycan conformations (“-gly” means with glycan).

NA	Monomer-bridged conformation	Standing Conformation	Dimer-bridged conformation
1NN2-gly	55.65%	38.65%	5.70%
2HTY-gly	5.75%	43.68%	50.57%
3NSS-gly	23.75%	50.02%	25.23%

### 146-N-glycan can block 150-cavity

**Fig 4:**
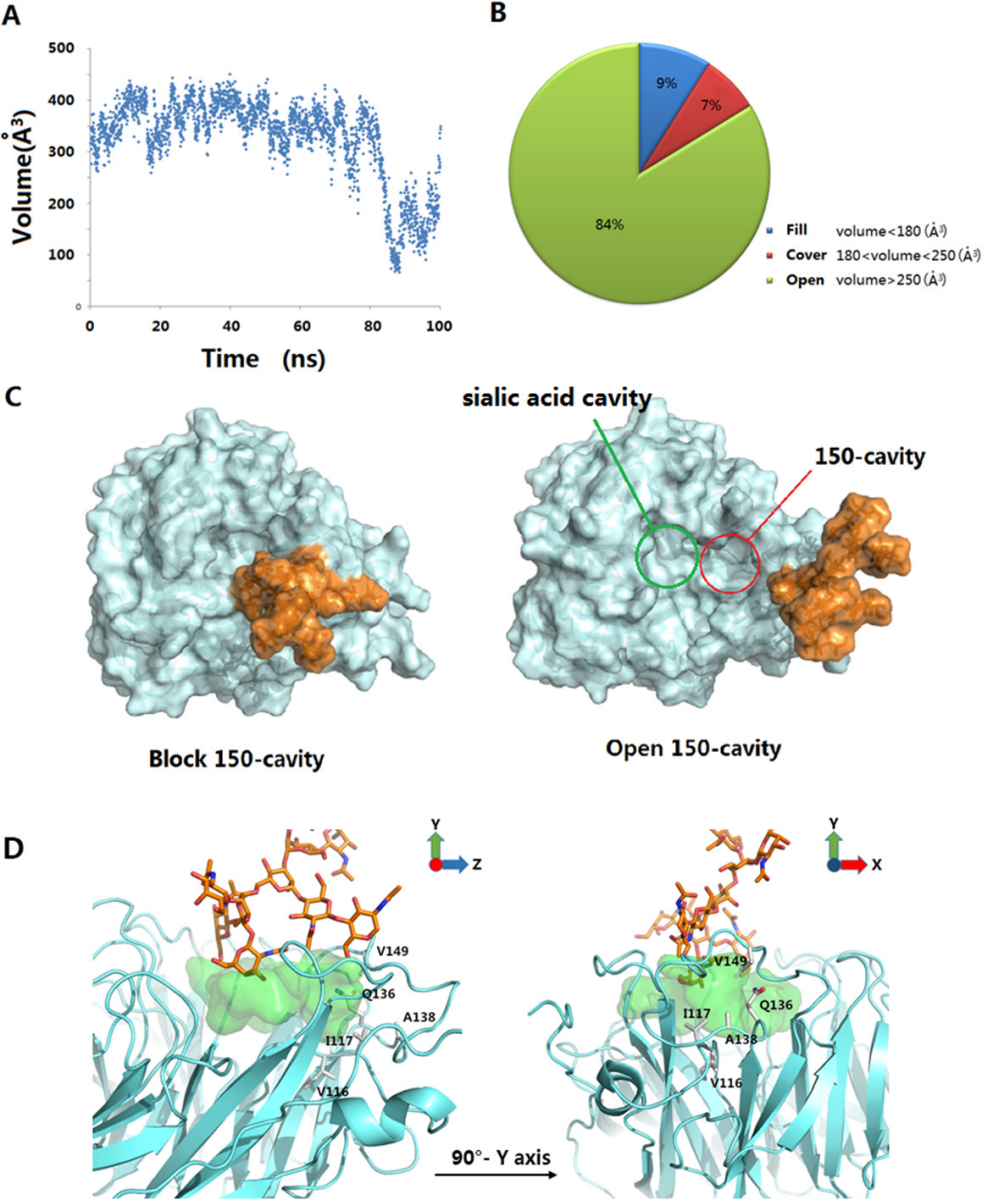
Block and open conformations of 146-N-glycan and the hydrophobic zone of 150-cavity. (A) 150-cavity volume of 2HTY chain A in 100 ns MD simulation. (B) The pie chart of three 146-N-glycan conformations clusters. (C) The blocking and opening 150-cavity conformations of 146-N-glycan. The 146-N-glycan was colored in orange. (D) The conformation of 146-N-glycan that covers NA substitute binding cavity. Residues V116, I117, Q136, A138 and V149 construct a hydrophobic zone of 150-cavity. The substitute binding cavity was colored in green and 146-N-glycan was colored in orange.

**Fig 5:**
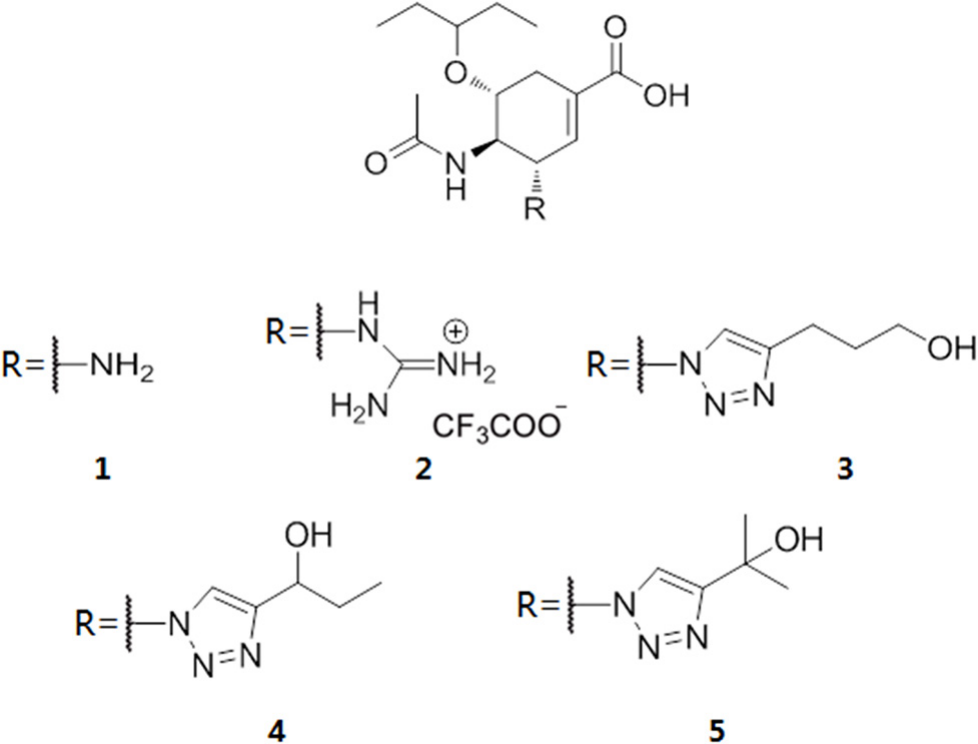
Five inhibitors that have co-crystal complex structure with N8 [26]

**Fig 6:**
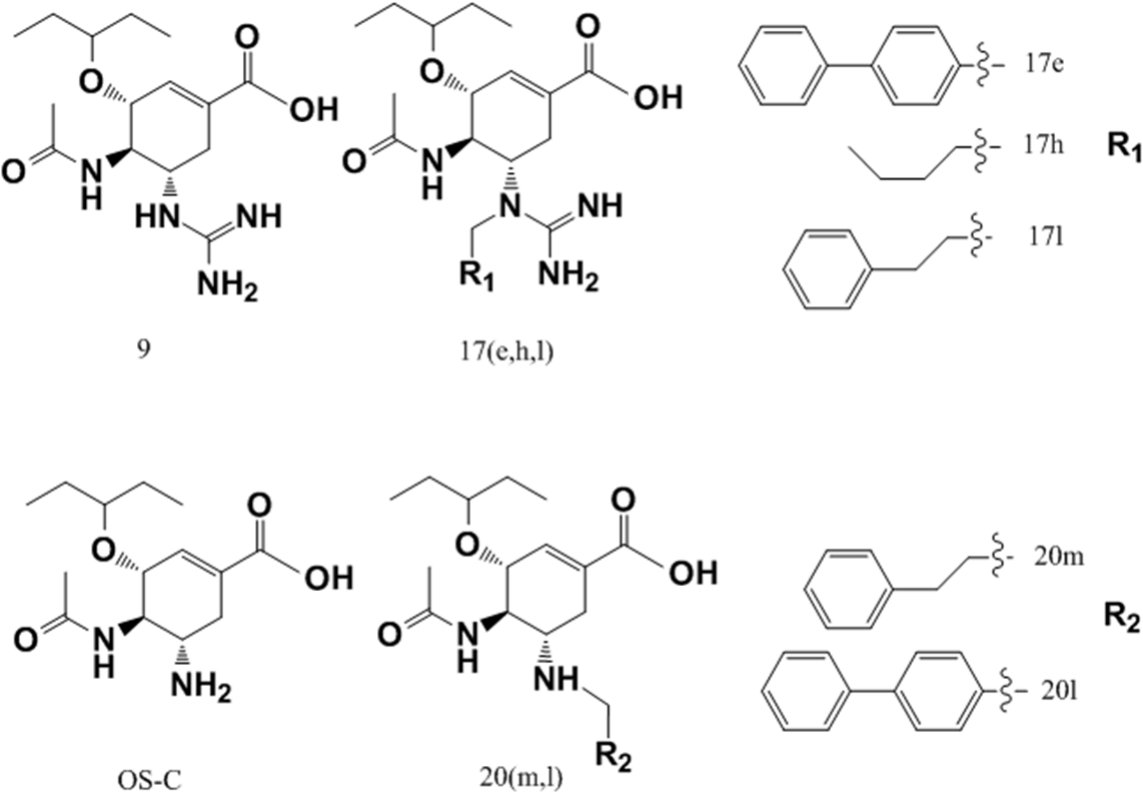
Seven inhibitors design to target 150-cavity. These inhibitors were come from Yuanchao Xie’s work [25]. oseltamivir carboxylate (OS-C) and oseltamivir derivatives (9, 17e, 17h, 17l, 20m, 20l). Inhibitors 9 and OS-C have no substituents bound to 150-cavity. Inhibitors 17h, 17l, 20m have flexible substituents while inhibitors 17e and 20l have rigid substituents.

**Fig 7:**
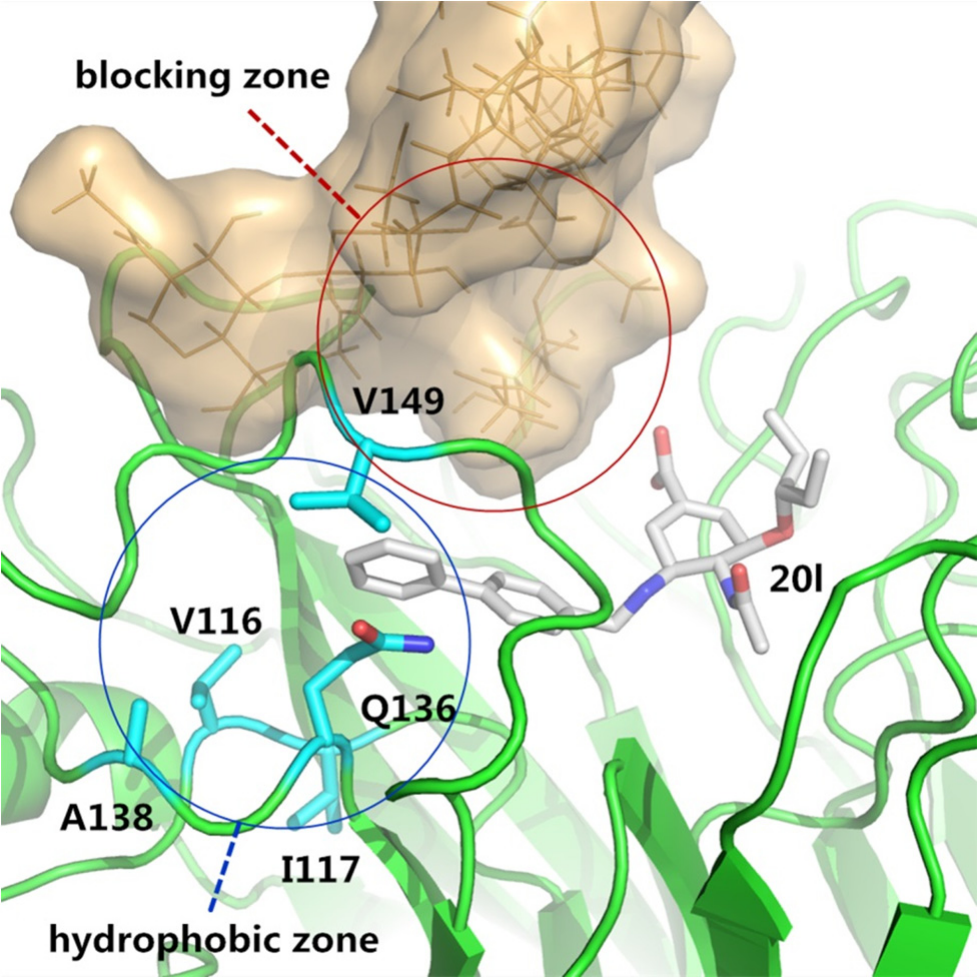
Conformation of Inhibitor 20l bind to N1 with 146-glycan. Conformation of N1 protein (green) come from crystal structure (PDB ID: 2HU0). 146-glycan, which was from 2HTY by superposition, was colored orange. Important residues V116, I117, Q136, A138 and V149, which construct the hydrophobic zone of 150-cavity were colored cyan. The long rigid substituent of 20l avoids binding to blocking zone of the 146-glycan and binds to the hydrophobic zone of 150-cavity.

In 2009, Zhenliang L. Wu’s work revealed that NA enzymatic activity of the 1918 flu increased after deglycosylation of N-glycan [22], which suggests that glycans may interfere with the binding of sialic acid and NA. Discovery of the 150-cavity then provided researchers with a new target for the design of novel inhibitors. However, the activities of all inhibitors that have been designed are considerably reduced compared with the activity of zanamivir [23–25]. Our simulations demonstrate that without 146-N-glycan, the portion comprised of the open 150-loop increases by approximately 20%, which may result in false-positive activity in inhibitors designed to target the 150-cavity. To determine whether 146-N-glycan can affect the binding of inhibitors targeting the 150-cavity, the 150-cavity volume in the A chain of 2HTY was calculated. The selection of the A chain of 2HTY was based on two considerations. First, the 150-loop in the 2HTY chain was maintained in the open confirmation throughout the entire MD simulation. Second, the 146-glycan of chain A did not interact with 146-glycans of adjacent chains during the entire MD simulation, thereby providing the 146-glycan the most flexibility. Fig 4A shows the 150-cavity volume from each snapshot extracted from the trajectory files obtained every 40 ps. The volume of the 150-cavity ranged from 50 to 450 Å^3^. The percentage of different 146-N-glycan conformations that influence the 150-cavity is presented in Fig 4B. The filled conformation (9%) represents the conformation in which 146-N-glycan occupies the 150-cavity, whereas the covered conformation (7%) represents the conformation in which the 146-N-glycan covers the entrance of the 150-cavity. Together, these two conformations are defined as the blocked conformation (Fig 4C), whereas the other conformation is defined as the open conformation. Based on these results, we hypothesized that the activities of inhibitors designed to target the 150-cavtiy might be affected by blocking the 146-N-glycan. To test the hypothesis, five inhibitors (Fig 5) were selected for the following study (inhibitors 1 and 2 only target orthosteric sialic acid binding sites, whereas compounds 3, 4, 5 also target the 150-cavity). These inhibitors form co-crystal structures when used to soak crystals of N8 [A/duck/Ukraine/1/63(H3N8)] (group-1 NA) [26], and they display experimental Ki values in vitro with H5N1 [23]. The binding energies of crystal conformations (S8 Fig) of inhibitors 1, 2, 3, 4, and 5 with NA protein (PDB ID: 4KS1, 4KS2, 4KS3, 4KS4, 4KS5) were calculated using Molecular mechanics with generalized Born and surface area solvation (MM-GBSA) with AMBER 12 for 2 ns MD simulation. As shown in Table 2, the calculated binding energies of inhibitors 3, 4 and 5 were similar to those of inhibitors 1 and 2, which indicates that all five inhibitors should exhibit similar inhibition activity. However, the experimental Ki values of inhibitors 1 and 2 (only binding to the sialic acid cavity) were considerably reduced compared with inhibitors 3, 4, and 5. One primary reason for the difference in activity between these two sets of inhibitors is that inhibitors 3, 4, and 5 might be blocked from binding to the entrance to the 150-cavity by the 146-N-glycan in 16% of conformations (as in Fig 4B). In contrast, for inhibitors 1 and 2, only a small portion of the entrance to the sialic acid cavity might be blocked by the 146-N-glycan. Furthermore, for inhibitors 3, 4, and 5, the most flexible inhibitor (inhibitor 3) displayed the lowest activity. This finding may be caused by the increased probability that a flexible substitute will interact with 146-N-glycan. Therefore, to prevent an inhibitor from interference with 146-N-glycan, the substitute should be longer and more rigid to reach the bottom of the 150-cavity where the hydrophobic residues V116, I117, A138 and V149 (Fig 4D) are located. Recently, in Yuanchao Xie’s work [25], two series of inhibitors were synthesized, and the ability of these molecules to inhibit H5N1 was verified by experiments. Seven inhibitors were selected from two series (Fig 6) from Yuanchao Xie’s work [25] and docked into the N1 protein (PDB ID: 2HU0). After a 2 ns MD simulation, the binding energies of the seven inhibitors were calculated using MM/GBSA in AMBER 12 (Table 2). The binding conformations are provided in S9 and S10 Figs. The results obtained are consistent with our conclusions. Table 3 shows that the calculated binding energies of inhibitors 17h, 17l, and 20m (with flexible substituents) do not correlate with their low experimental activities. Alternatively, inhibitors 17e and 20l (with rigid substituents), which may not interact with the 146-glycan site, exhibit better experimental activities. Inhibitors 20l, which can interact with the hydrophobic zone of the 150-cavity (shown in Fig 7), exhibits a particularly high activity (IC_50_ = 1.9 nm). Based on this model, we suggest that to avoid being blocked by the 146-N-glycan, inhibitors targeting the 150-cavity should have long, rigid substituents that can bind to the hydrophobic zone, which is surrounded by residues V116, I117, A138 and V149 of the 150-cavity. Furthermore, in 1993, Shengqiang Li’s work [27] revealed that NA activity of Influenza A/WSN/33 can be reduced by 10-fold by a glycosylate at position 130 (corresponding to the 146-N-glycan of 3NSS). This result indicates that if the 146-N-glycan is shifted to block the entrance of the NA active site, it can reduce the activity of NA. Therefore, it may be useful to design compounds to prevent the 146-N-glycan from forming a dimer-bridge to provide more standing conformations. Such a compound could represent a new type of NA inhibitor.

**Table 2:** Experimental^a^ and calculated binding energies^b^ of the five NA inhibitors.

Inhibitor ID	PDB ID	Experimental Ki^*a*^ (nM)	Calculated binding Energy^*b*^ (kcal/mol)
1	4KS1	1.5	-33.8±2.4
2	4KS2	0.46	-47.7±4.1
3	4KS3	460	-55.9±3.8
4	4KS4	72	-32.3±2.2
5	4KS5	130	-32.8±3.6

^*a*^ Experimental Ki data in Mohan S’s work [23] was used in this table.

^*b*^ MM/GBSA in AMBER 12 was used to calculated the binding energy.

**Table 3:** Experimental^a^ and calculated binding energies^b^ of the seven NA inhibitors.

Inhibitor ID	Name	NA inhibition assayIC_50_ ^*a*^ (nM)	Calculated binding Energy^*b*^ (kcal/mol)
6	9	6.9	-37.3±2.7
7	17e	150	-58.4±4.5
8	17h	780	-44.1±3.8
9	17l	790	-57.8±4.1
10^*c*^	OS-C	17	-25.0±3.1
11	20m	32	-33.5±3.0
12	20l	1.9	-32.4±3.3

^*a*^ Experimental IC_50_ data in Yuanchao Xie’s work [25] was used in this table.

^*b*^ MM/GBSA in AMBER 12 was used to calculated the binding energy.

^*c*^ Binding conformation of OS-C is the co-crystal conformation (PDB ID: 2HU0).

## Conclusion

Our MD simulations highlight the important effect of the 146-N-glycan on the 150-cavity of N1 and N2 systems across a dynamic ensemble of NA proteins. To our knowledge, this is the first study to employ long time-scale MD simulations of NA tetramer proteins complexed with glycan. Our simulation revealed that the 146-N-glycan could stabilize predominant l50-loop conformations and prevent conformational transformation in both N1 and N2. In particular, when simulating the 09N1 protein 3NSS, simulations with glycan showed the molecule to have the opposite preferential conformation to that without glycan. Cluster analysis of the MD trajectories captured three distinct conformational clusters of glycan on proteins: monomer-bridged, dimer-bridged, and standing conformations, which correlate to the closed, open, and open+closed forms of 150-loop, respectively. These conformations therefore control the volume of the 150-cavity. Our simulations also revealed that in a small portion of conformations, glycan can block the 150-cavity. This finding could be a primary reason that existing inhibitors targeting the 150-cavity exhibit lower bio-activities relative to those that bind to the original sialic binding cavity. Based on this model, we suggest that to avoid being blocked by the 146-N-glycan, inhibitors targeting the 150-cavity should have long, rigid substituents that can bind to the hydrophobic zone, which is surrounded by residues V116, I117, A138 and V149 of the 150-cavity. Furthermore, we propose that compounds designed to prevent 146-N-glycan from forming a dimer bridge might result in a greater proportion of the 146-N-glycans in the standing conformation, which could reduce the probability of binding to sialic acid. Molecules designed in this manner could represent a new form of NA inhibitor.

## Methods

### Preparation of the initial structures

Six simulation systems were created and designated as 3NSS-No-Glycan, 3NSS-With-Glycan, 2HTY-No-Glycan, 2HTY-With-Glycan, 1NN2-No-Glycan and 1NN2-With-Glycan. The atomic coordinates of 3NSS–No-Glycan and 2HTY-No-Glycan were extracted from 3NSS and 2HTY, respectively, by removing the glycans. The atomic coordinates of 1NN2-With-Glycan were extracted from 1NN2, whereas the atomic coordinates of 1NN2-No-Glycan were extracted from 1NN2 by removing the glycans. The glycans of 3NSS-With-Glycan and 2HTY-With-Glycan were created using the corresponding atomic coordinates of the fractional 146-N-glycan in 3NSS as a template, using a complex glycan structure type command that was obtained from the Glyprot Server (http://www.glycosciences.de/modeling/glyprot/) [28].

The structure of the NA tetramer was created from monomer structures by applying a VMD 1.9 matrix transformation using Tcl script [29]. Histidine residues were protonated at pH 6.5 using the PDB2PQR server 6 [30]. Disulfide linkages were labeled with the proper AMBER notation and the resultant files were processed using XLeap from the AMBER 12 [20] program, using the AMBER99SB and the GLYCAM06 force fields. A truncated octahedral box of pre-equilibrated TIP3P water was added to solvate each system with a pad of 10 Å. Counter ions were added to neutralize each system.

### Molecular dynamics simulations

MD simulations of all systems were conducted using a version of the PMEMD module from AMBER 12. For each system containing glycans at position N146, the minimization and equilibration procedures were as follows. To maintain the glycan direction as it appears in the crystal structure, the structures were minimized in four stages. First, glycans and protein were all restrained. Second, the first three glycosyl units of the glycans and the protein were restrained. Third, only the protein was restrained. Finally, all systems were unrestrained. The restraints were reduced in the four stages over 20000 steps of steepest descent and conjugate gradient minimization.

After minimization, the system was gradually heated from 0 K to 300 K in a canonical constant volume ensemble using Langevin dynamics with the collision frequency gamma_ln = 2.0 and with position restraints on the solute molecules. The pressure was then equilibrated in five steps at 300 K in the NPT ensemble, during which a decreasing harmonic force constraint on the 146-glycans of 50, 25, 10, 5 and 1 kcal•mol^-1^•Å^-1^ was applied at a target pressure of 1 atm. Then, 1 ns of NPT MD simulation without restraints was run before production runs at 300 K using Langevin dynamics with the collision frequency gamma_ln = 2.0. Finally, 100 ns MD simulations for each system were conducted at 300 K and at 1.0 atm. of pressure. Electrostatics were handled using the particle mesh Ewald (PME) algorithm with a 10.0 Å direct space non-bonded cutoff. All bonds involving hydrogen atoms were constrained using the SHAKE algorithm with a time step of 2.0 fs. The coordinates of the trajectories were saved every 10 ps during the entire MD run.

### Docking

The protein structure (2HU0) was downloaded from the PDB Databank as a receptor. Autodock-vina was applied to dock the inhibitors into the receptor, with residue R156 being defined as flexible. Exhaustiveness was set at 16. The optimal docking conformation of inhibitor 20l was defined as a reference conformation. Finally, conformations that had the same binding mode as the reference conformation were selected for binding energy calculations for the remainder of the five inhibitors.

### Binding energy calculations

The ligand binding energies and the interaction energies between the 146-N-glycan and the NA tetramer were calculated using MM/GBSA [30]. For calculations of the interaction energy, only the E_vdw_ and E_coul_ were considered. For calculations of the ligand binding energy, E_MM_ and G_solv_ were considered. The entropy contributions were neglected because the same receptor was used, and the normal mode calculations are computationally expensive and introduce errors that cause significant uncertainty in the results.

$$\Delta G=\Delta E_{MM}+{\Delta G}_{solv}-T\cdot \Delta S={\Delta E}_{bat}+{\Delta E}_{vdw}+{\Delta E}_{coul}+{\Delta G}_{solv.p}+{\Delta G}_{solv.np}-T\cdot \Delta S$$ (1)

where E_MM_ is a gas-phase MM energy. The free energy for each species (ligand, receptor, and complex) was decomposed into a gas-phase MM energy that contained polar and nonpolar solvation terms and an entropy term (which was neglected). E_MM_ comprises E_bat_ (the sum of bond, angle, and torsion terms in the force field), a Van der Waals term, E_vdw_ and a coulombic term, E_coul_. G_solv.p_ is the polar contribution to the free energy of solvation, often computed via the Generalized-Born (GB) approximation. G_solv.np_ is the nonpolar free energy of solvation, usually computed as a linear function of the solvent-accessible surface area (SASA). A 2 ns MD simulation was performed on each complex. The final 50 snapshots with an interval of 10 ps were extracted to calculate the binding energy.

### Volume calculation

The volume of the 150-cavity was calculated using the POVME1.0 program [31]. A 3D-grid with 0.5 Å spacing that covered the 150-cavity was created in the initial structure (see S11 Fig). Snapshots were extracted from the trajectory files every 40 ps and superimposed onto the reference structure (the initial crystal structure). Grid points overlapping with the protein atoms in the snapshots were systematically removed. The volume of the 150-cavity was then calculated by counting the remaining grid points.

### Cluster analysis

To identify the structural representations of the 150-loop conformations that were generated by the MD simulations, cluster analysis was performed using cpptraj in AMBER 12 [20]. Snapshots were clustered every 10 ps using an average-linkage [32] clustering algorithm with an epsilon value of 2.0.

To reveal the relationship between the conformations of the 150-loop and 146-N-glycan, the conformers from the MD snapshots were projected onto the essential space (RMSD and dihedral) [33], and the molecular potential was given by Eq (2) [34].

E=−RTln(ρ) (2)

where E represents the molecular potential, ρ is the conformer density and RT is the thermal energy.

Three local minima of energies were selected and the relative conformations of 146-N-glycan were selected as representative cluster conformations. Finally, cluster analysis was performed.

## Supporting Information

S1 Fig Root mean square deviation (RMSD) of six systems in coordinates of alpha-carbon as a function of the simulation time. (TIF)

S2 Fig B-factor comparison between 1NN2 crystal structure and 1NN2 MD simulation results. (TIF)

S3 Fig B-factor comparison between 2HTY crystal structure and 2HTY MD simulation results. (TIF)

S4 Fig B-factor comparison between 3NSS crystal structure and 3NSS MD simulation results. (TIF)

S5 Fig The selectivity of inhibitor 20l. (TIF)

S6 Fig Plane defined by S145: CA, L223: CA, P301: CA to evaluate the dihedral of 146-glycan. (TIF)

S7 Fig The interaction energy between each glycan and the other parts of tetramer of 3NSS versus the dihedral angle of glycans. (TIF)

S8 Fig Crystal complex structure 4KS1~4KS5 of ligand 1~5 binding to N8. (TIF)

S9 Fig Docking conformations of ligands 9, 17e, 17h, and 17l. (TIF)

S10 Fig Docking conformations of ligands OS-C, 20l and 20m. (TIF)

S11 Fig The volume probe sphere in the 150-cavity generated by POVME1.0. (TIF)

S1 Table Activities of inhibitors of 6~12 to H5N1-1220 and H9N2-S2 (DOCX)

S1 Text Calculation and Statistics of interaction Energy between each glycan and the other part of complex (DOCX)
