# Supplementary figures and images for: The Mechanism by which 146-N-Glycan Affects the Active Site of Neuraminidase
Source: PLoS One. 2015 Aug 12;10(8):e0135487. doi: 10.1371/journal.pone.0135487 (PMC4534095; doi:10.1371/journal.pone.0135487)

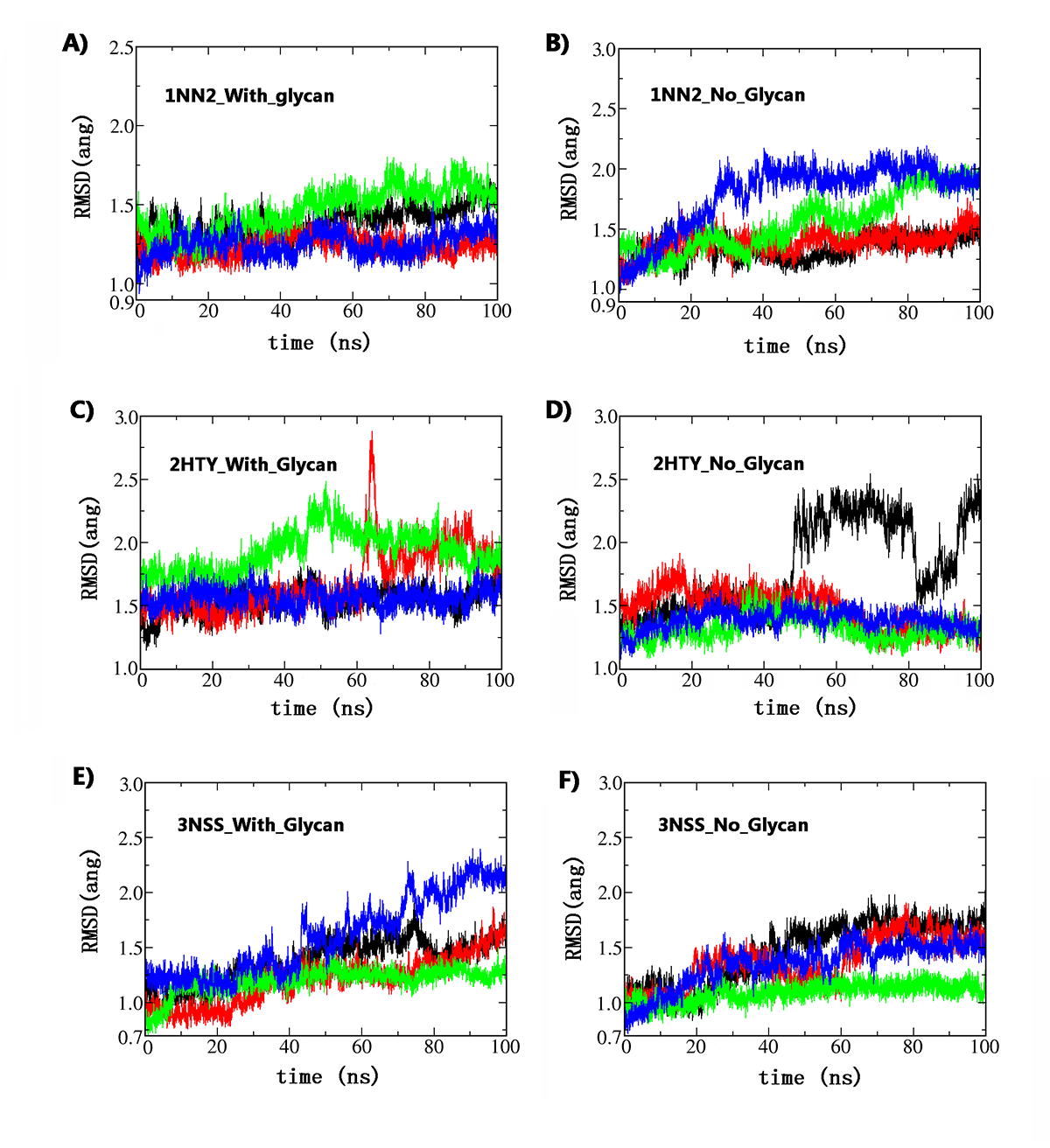

Supplement: S1 Fig — (TIF) [file pone.0135487.s001.tif]

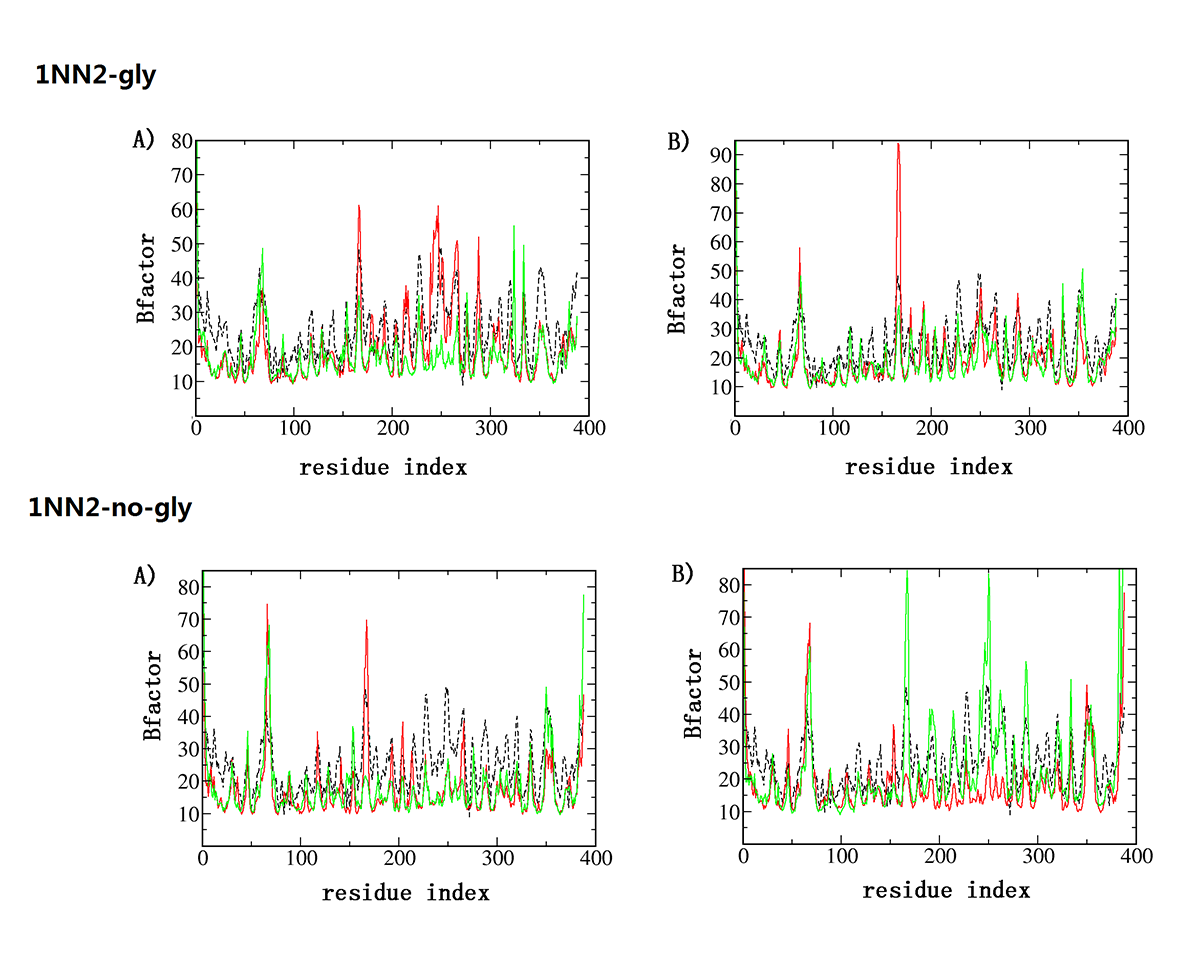

Supplement: S2 Fig — (TIF) [file pone.0135487.s002.tif]

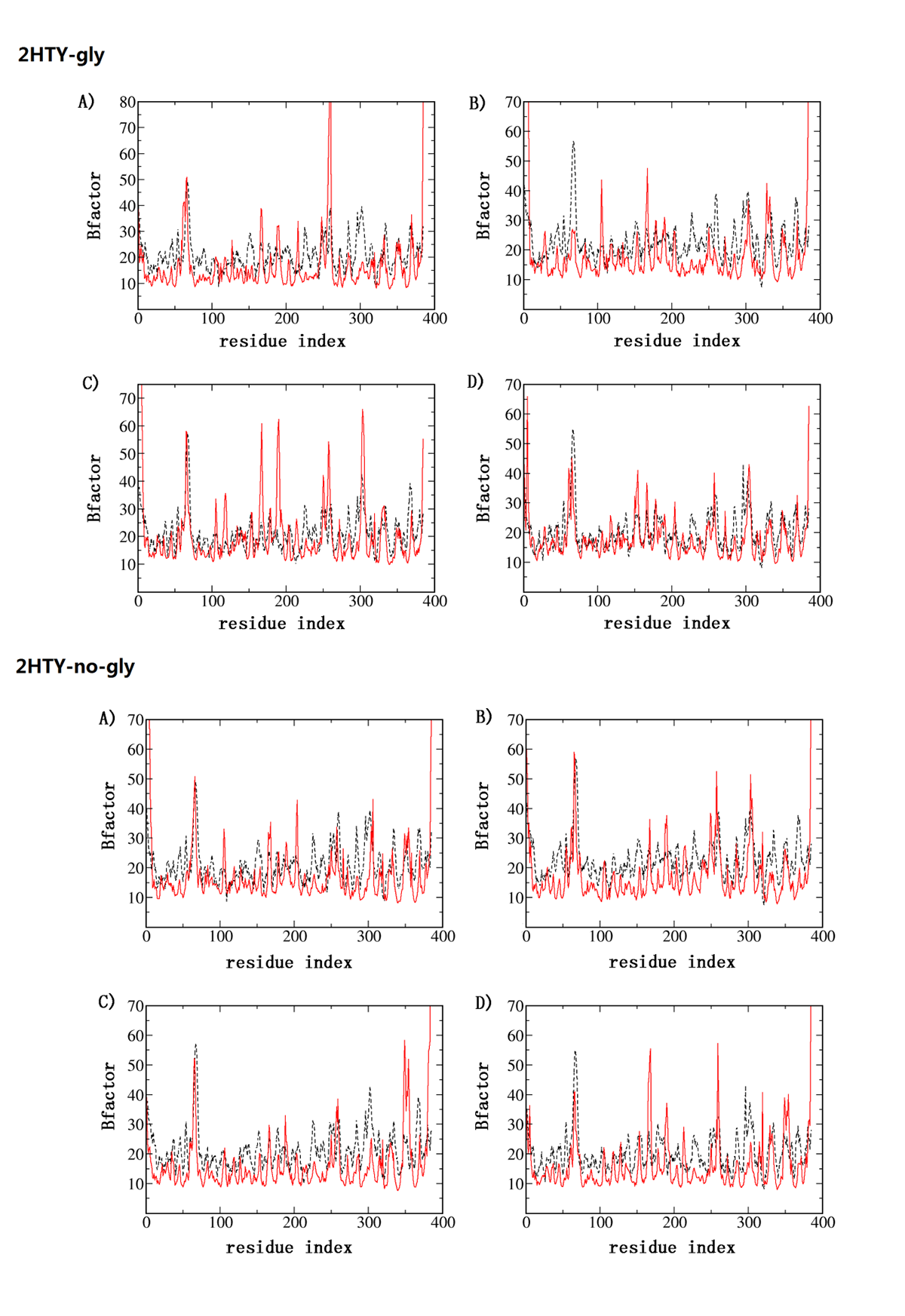

Supplement: S3 Fig — (TIF) [file pone.0135487.s003.tif]

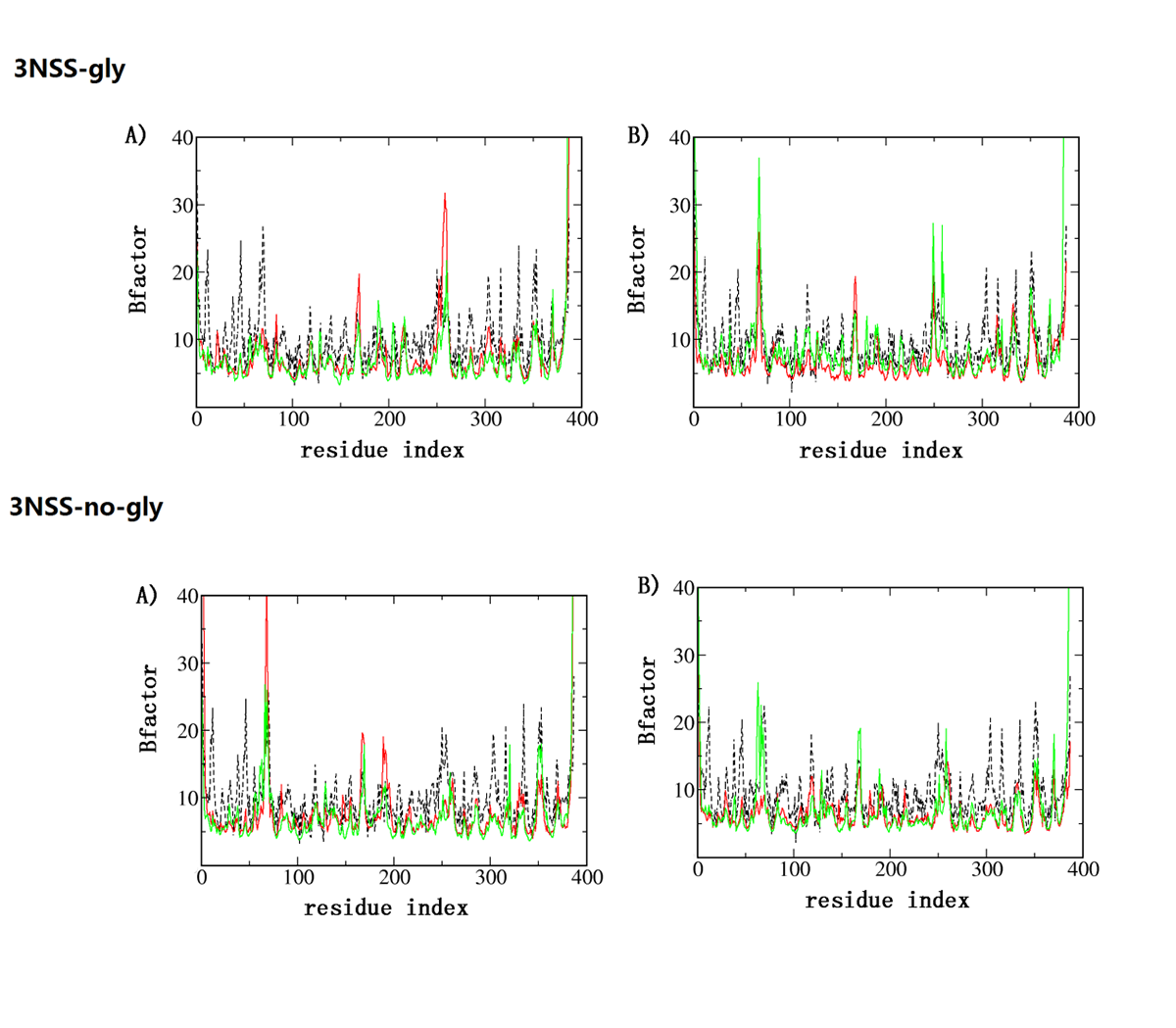

Supplement: S4 Fig — (TIF) [file pone.0135487.s004.tif]

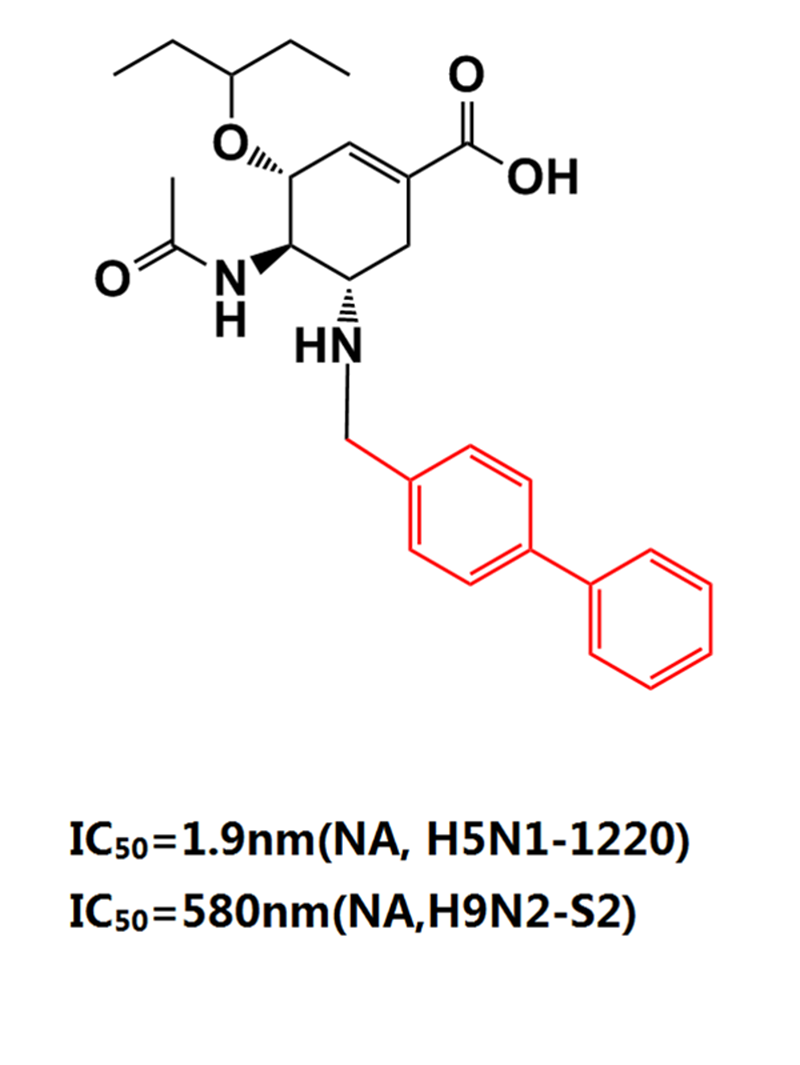

Supplement: S5 Fig — (TIF) [file pone.0135487.s005.tif]

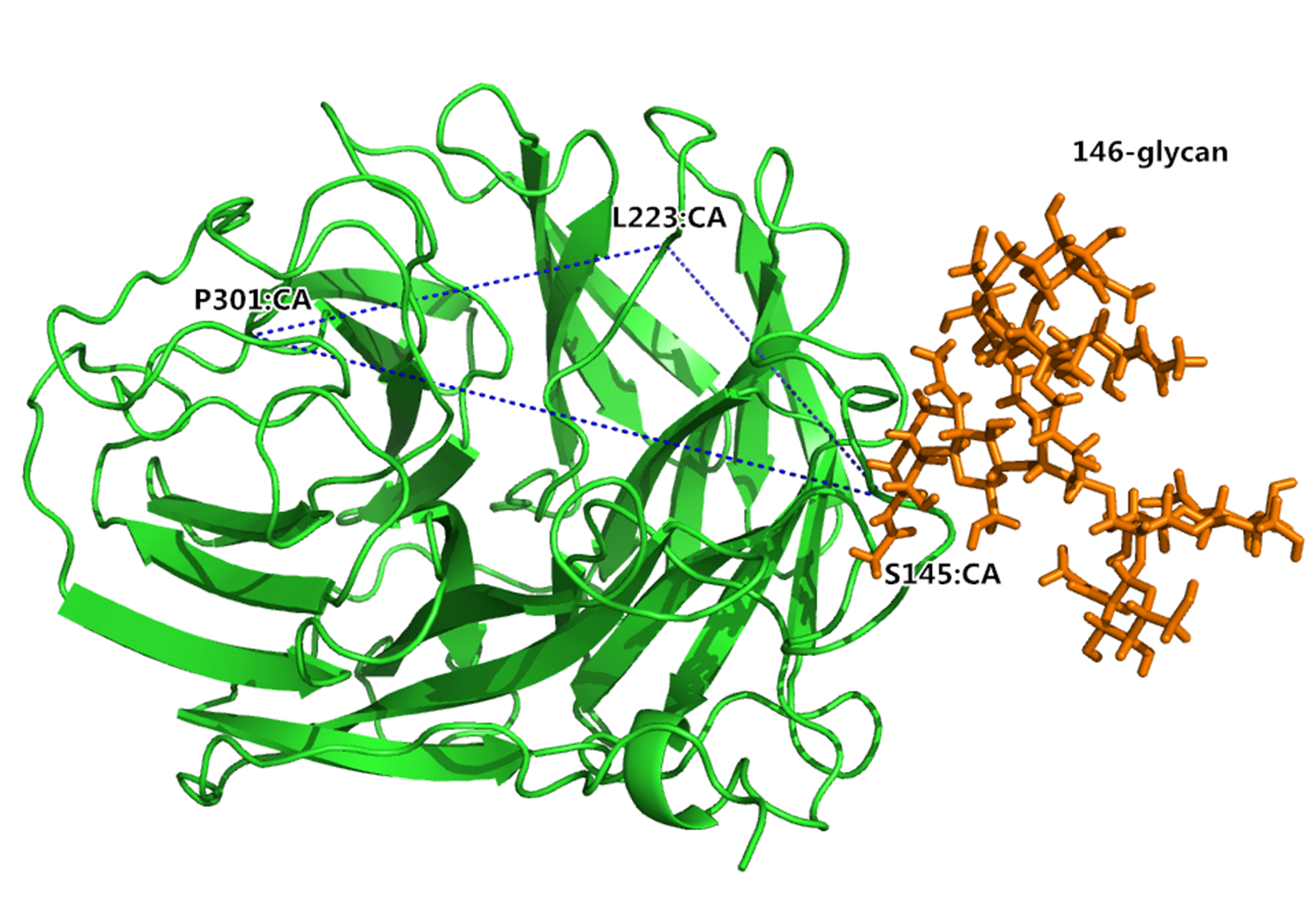

Supplement: S6 Fig — (TIF) [file pone.0135487.s006.tif]

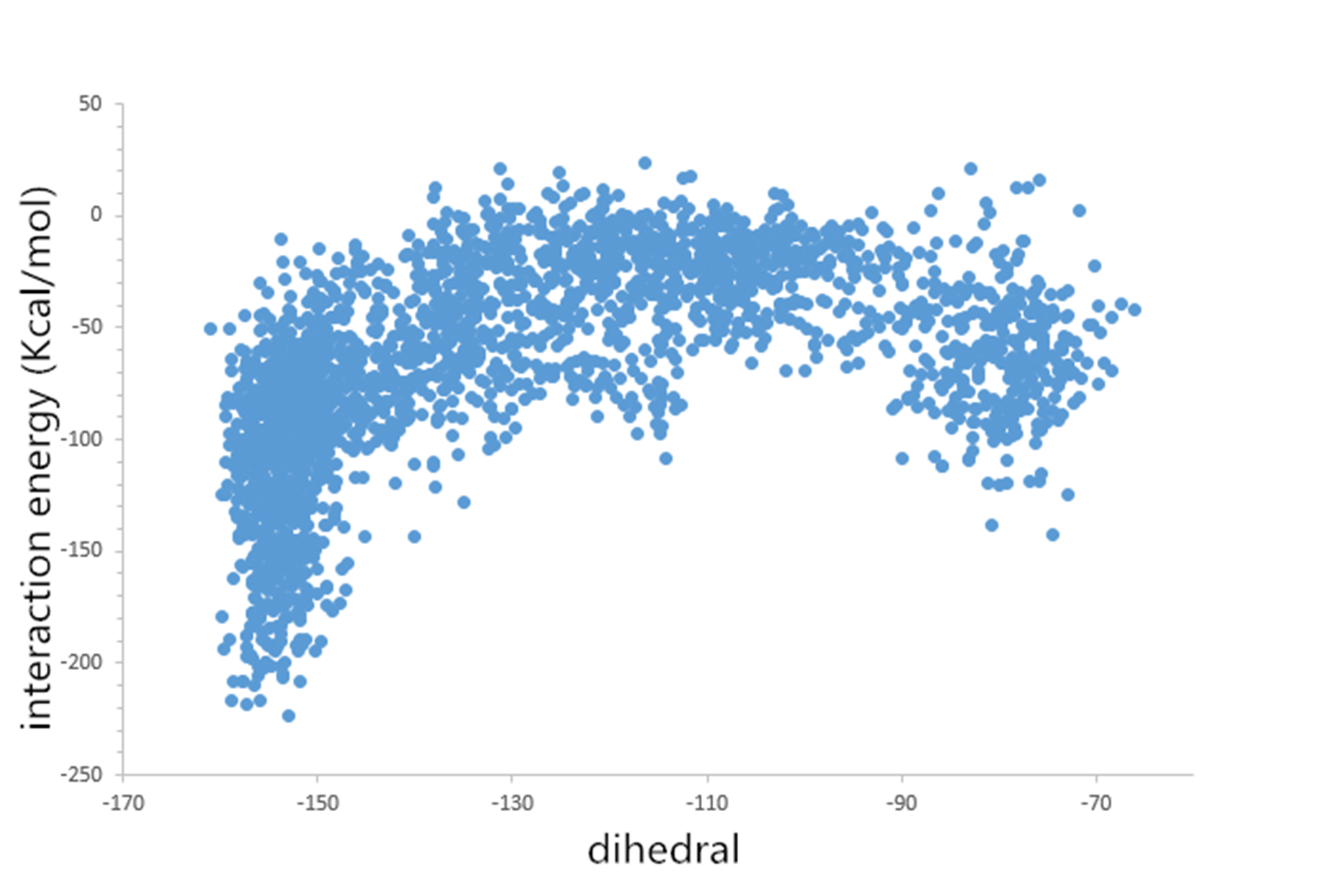

Supplement: S7 Fig — (TIF) [file pone.0135487.s007.tif]

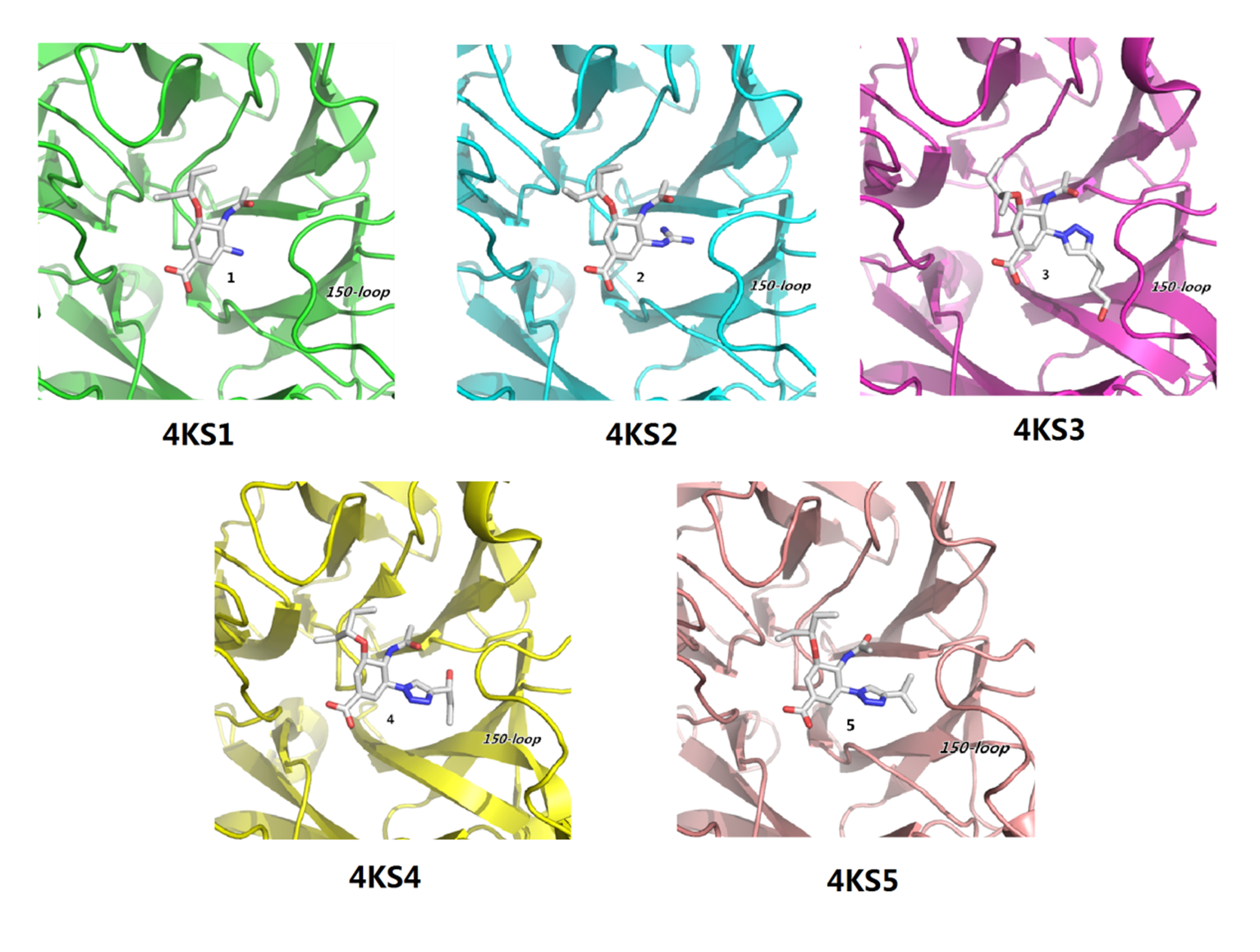

Supplement: S8 Fig — (TIF) [file pone.0135487.s008.tif]

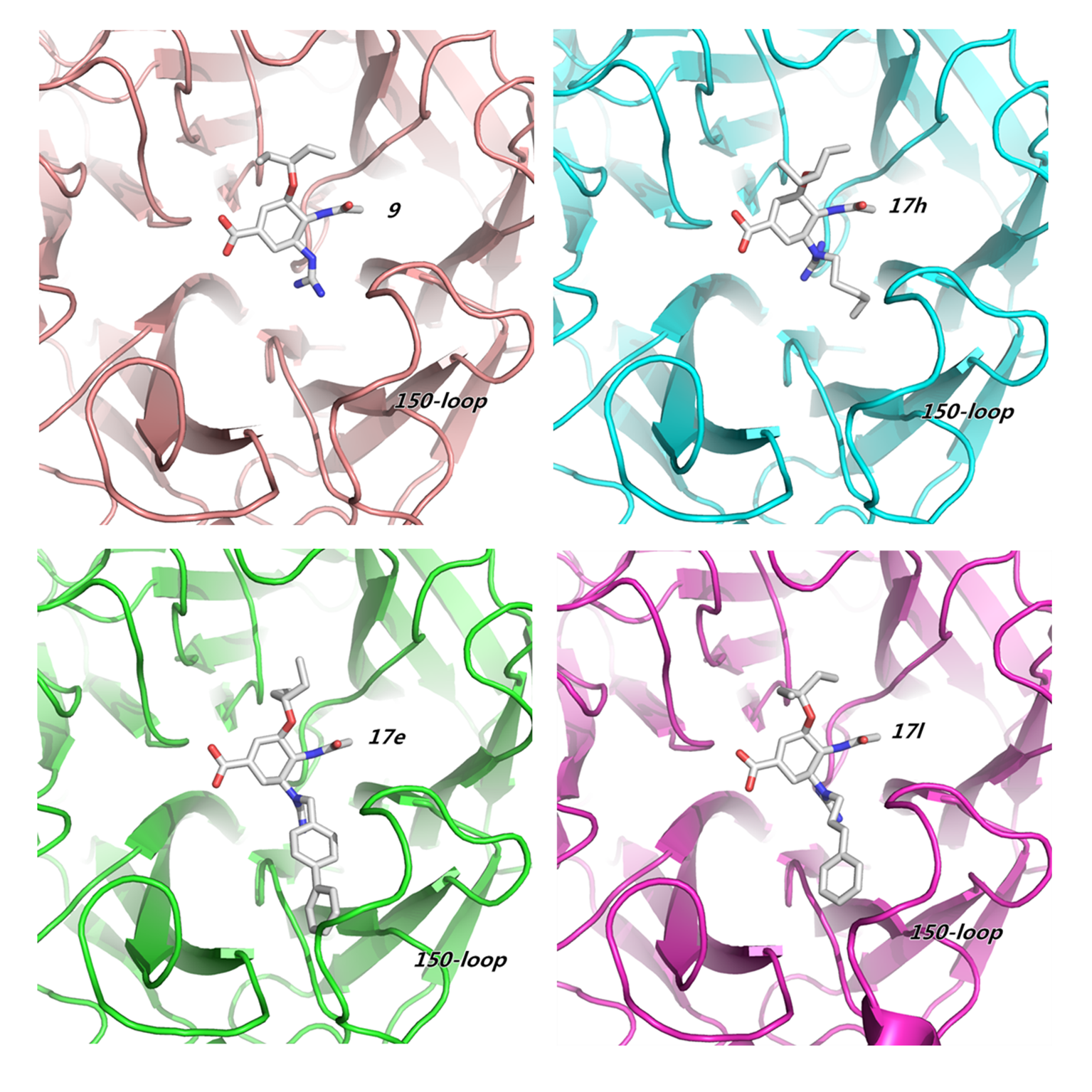

Supplement: S9 Fig — (TIF) [file pone.0135487.s009.tif]

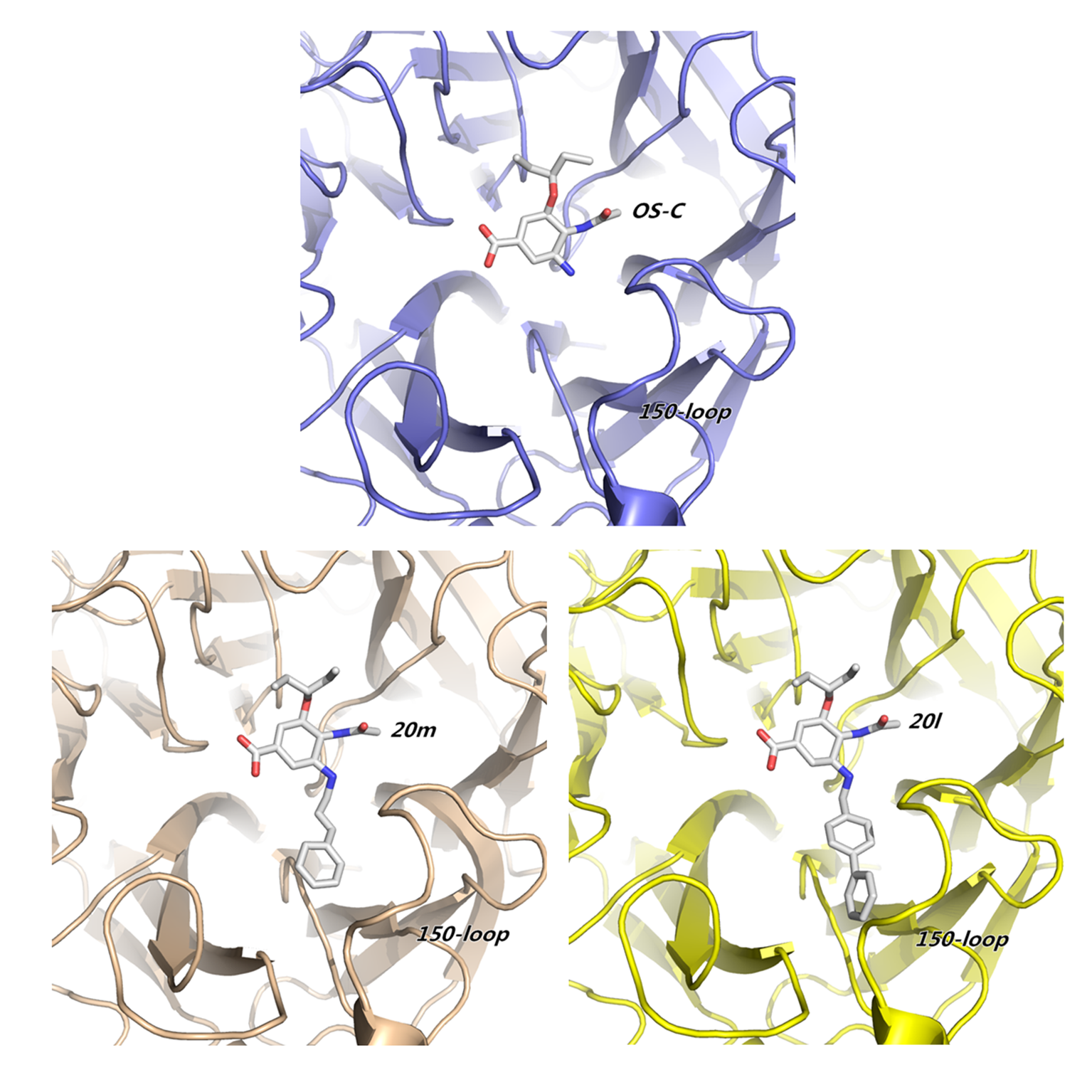

Supplement: S10 Fig — (TIF) [file pone.0135487.s010.tif]

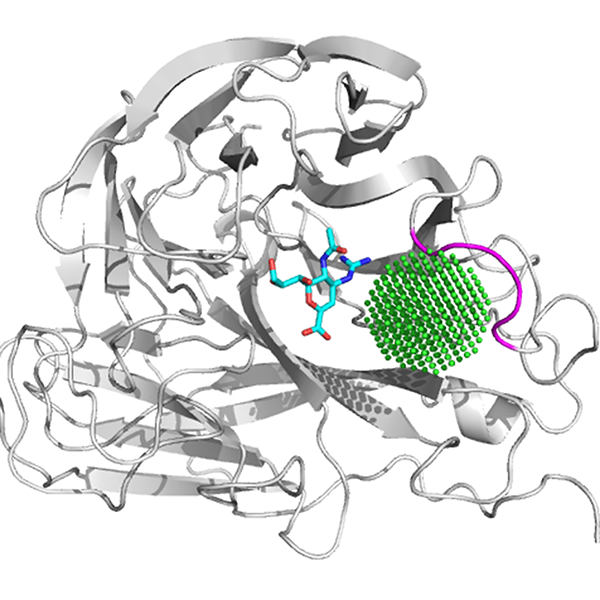

Supplement: S11 Fig — (TIF) [file pone.0135487.s011.tif]
