# Supplementary material for: The Mechanism by which 146-N-Glycan Affects the Active Site of Neuraminidase
Source: PLoS One. 2015 Aug 12;10(8):e0135487. doi: 10.1371/journal.pone.0135487 (PMC4534095; doi:10.1371/journal.pone.0135487)
Supplement: S1 Table — (DOCX) [file pone.0135487.s012.docx]

**S1 Table. Activities*^a^* of inhibitors of 6~12 to H5N1-1220 and H9N2-S2**

| Inhibitor ID | Name | H5N1-1220  IC_50_ (μM) | H9N2-S2  IC_50_ (μM) |
| --- | --- | --- | --- |
| 6 | 9 | 0.0069±0.001 | 0.011±0.001 |
| 7 | 17e | 0.15±0.018 | 0.37±0.045 |
| 8 | 17h | 0.78±0.075 | >5.0 |
| 9 | 17l | 0.79±0.087 | >5.0 |
| 10 | OS-C | 0.017±0.001 | 0.0031 |
| 11 | 20m | 0.032±0.006 | >5.0 |
| 12 | 20l | 0.0019 | 0.58±0.11 |

*^a^* Experimental IC_50_ data in Yuanchao Xie’s work [26] was used in this table
