# Supplementary material for: The Mechanism by which 146-N-Glycan Affects the Active Site of Neuraminidase
Source: PLoS One. 2015 Aug 12;10(8):e0135487. doi: 10.1371/journal.pone.0135487 (PMC4534095; doi:10.1371/journal.pone.0135487)
Supplement: S1 Text — (DOCX) [file pone.0135487.s013.docx]

**S1 Text. Calculation and Statistics of interaction Energy between each glycan and the other part of complex**

The interaction Energy carried out with AMBER 12 on snapshot of every 160 ps in trajectory of 3NSS. To remove the influence of the first Glycosylation that link the glycan to NA protein, it was not count in the calculation. The interaction energy of remain nine glycosylation of each glycan and other part of complex was calculated with the following Eq. (1). Total 2500 interaction energy were plot versus the dihedral angel of between plane A defined by mass center of glycan, S145:CA, L223:CA and plane B defined by S145:CA, L223:CA, P301:CA (Figure S6). The reason to select these three atoms is that they have lower B-factor which means they can give a relative stable reference plane. Then, the average interaction energy of each dihedral angle degree was calculated by averaging the points drop in each degree (Fig. 3). Dimer-bridge，standing，monomer-bridge conformations were defined by dihedral angle φ <-140, -140<φ<-90, φ>-90.

$$E_{interaction}=E_{complex}{-E}_{glycan}-E_{other} =\left( E_{vdw\left( complex \right)}+E_{ele\left( complex \right)}+E_{int\left( complex \right)} \right)-\left( E_{vdw\left( glycan \right)}+E_{ele\left( glycan \right)}+E_{int\left( glycan \right)} \right) -\left( E_{vdw\left( other \right)}+E_{ele\left( other \right)}+E_{int\left( other \right)} \right) (1)$$
